# Human cognitive performance-associated gut microbiota influences learning and memory in mice through arginine and pterin metabolism

**DOI:** 10.1080/19490976.2026.2750588

**Published:** 2026-11-10

**Authors:** Harry Tracey, Anna Antolín, Joan Teichenne, Oliver A. H. Jones, Thi Thu Hao Van, Josep Puig, Rafel Ramos, Josep Garre-Olmo, José Manuel Fernández-Real, Jordi Mayneris-Perxachs, Josep M. Del Bas

**Affiliations:** a Eurecat, Centre Tecnològic de Catalunya, Unitat de Nutrició i Salut, Reus, Spain; b Integrative Systems Medicine and Biology Group, Girona Biomedical Research Institute (IDIBGI-CERCA), Salt, Spain; c School of Science, RMIT University, Victoria, Australia; d Department of Medical Sciences, School of Medicine, University of Girona, Girona, Spain; e CIBER Centro de Investigación Biomédica en Redde Fisiopatología de la Obesidad y Nutrición (CIBERobn), Madrid, Spain; f Department of Radiology (CDI) and IDIBAPS, Hospital Clínic de Barcelona, Barcelona, Spain; g Vascular Health Research Group of Girona (ISV-Girona), (Institut Universitari per a la Recerca en Atenció Primària Jordi Gol I Gorina -IDIAPJGol), Red de Investigación en Cronicidad, Atención Primaria y Promoción de la Salud -RICAPPS- ISCIII, Jordi Gol Institute for Primary Care Research, Girona, Spain; h Research in Vascular Health Group, Girona Biomedical Research Institute (IDIBGI-CERCA), Salt, Spain; i Department of Nursing, University of Girona, Girona; j Research Group on Health, Gender and Aging, University of Girona, Girona; k Department of Diabetes, Endocrinology and Nutrition, Dr. Josep Trueta University Hospital, Girona, Spain; l Nutrition, Eumetabolism and Health Group, Girona Biomedical Research Institute (IDIBGI-CERCA), Girona, Spain; m Department of Biochemistry and Biotechnology, Nutrigenomics Research Group, Universitat Rovira i Virgili, Tarragona, Spain; n Institut de Recerca Biomèdica Catalunya Sud, Hospital Universitari Joan 23 de Tarragona, Tarragona, Spain; o Center of Environmental, Food and Toxicological Technology—TecnATox, Rovira i Virgili University, Reus, Spain

**Keywords:** Ageing, cognitive decline, gut–brain axis, symbol digit modalities test, multi-omics

## Abstract

The human gut microbiome undergoes changes across the lifespan and has been implicated in age-related decline. The symbol digit modalities test (SDMT) is a measure of information processing speed and is sensitive to mild cognitive impairment. Here, we assessed SDMT scores and fecal shotgun metagenomics in older individuals from the Imageomics cohort (*n* = 867) and performed fecal microbiota transplantation (FMT) from human donors to male C57BL/6 mouse recipients. In mice, we evaluated cognitive performance using the Morris water maze (MWM) and measured neurone density, fecal shotgun metagenomics, hippocampal metabolomics, and cortical gene expression to identify microbial-derived functional properties related to cognitive performance. In humans, both microbial species composition and functional profiles were associated with cognitive performance. FMT from high-SDMT donors improved cognitive performance and increased neuronal density in recipient mice compared with FMT from low-SDMT donors. In both humans and mice, higher cognitive performance was associated with arginine and proline metabolism and pterin-related pathways. In mice receiving microbiota from high-SDMT donors, cortical gene expression showed enrichment of dopamine receptor pathways and reduced dopamine reuptake and catecholamine biosynthesis. Our findings highlight the potential role of the gut microbiome in cognitive performance among older individuals, particularly information processing speed, and identify potential mechanisms involving alterations in arginine and pterin metabolism. Integrating human and mouse data supports a model in which microbiota-associated metabolic remodeling influences cognitive performance.

## Graphical abstract

**Figure:**
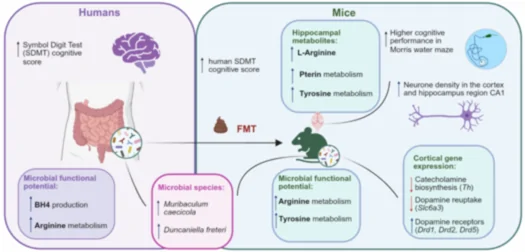

## Introduction

With an increasing ageing population, strategies to preserve cognitive function in later life are increasingly important for healthcare systems and society.^1^ Ageing is accompanied by widespread structural changes in the brain, including overall cerebral atrophy, ventricular enlargement, increased white matter hyperintensities, and changes in white matter microstructure.^2‐4^ The hippocampus plays a central role in learning, memory, and neural plasticity and is particularly vulnerable to age-related structural and functional changes.^5^ The symbol digit modalities test (SDMT) is used to detect cerebral dysfunction and mild cognitive impairment.^6-8^ The SDMT measures information processing speed and visual learning and processing, and requires intact activation of the frontal, parietal, occipital, and cerebellar regions.^8‐11^ Lower SDMT scores have been strongly associated with brain atrophy in neurological disorders such as multiple sclerosis. The SDMT has also demonstrated clinical utility in identifying cognitive impairment across different clinical populations, including traumatic brain injury, mild cognitive impairment, and dementia. More recently, poorer SDMT performance has been associated with greater amyloid-*β* deposition, a key marker of Alzheimer's disease pathology.^12‐14^

The human gut microbiome undergoes substantial changes during ageing, and this has been implicated in age-related decline.^15^ Changes in microbial composition and function can alter metabolic, inflammatory, and neurological processes.^16‐18^ Age-related alterations in the gut microbiome are highly variable between individuals and may be influenced by multiple factors, including physiological changes, health status, diet, environmental exposures, and host genetics.^19^ ^,^ ^20^ The gut microbiota can also contribute to the host's health during ageing. The concept of “unhealthy ageing” is characterized by an earlier decline in protective commensal taxa accompanied by increased pathobionts, inflammation, frailty, and degeneration.^15^ In contrast, “healthy ageing”, often observed in centenarians free from major disease, is associated with preserved cognitive function and reduced levels of chronic inflammation.

Microbial-derived metabolites are known to influence cognition during ageing.^21^ The gut microbiota influences cognitive function through the microbiota‒gut‒brain axis. This refers to the bidirectional communication between the microbiota, gut, and brain. Here, the gut microbiota can interact with the brain via the nervous, endocrine, immune, and systemic circulatory systems.^22^

Although increasing evidence links the gut microbiota and metabolic activity to several cognitive domains^16^ ^,^ ^17^ and mental health,^23^ ^,^ ^24^ it remains unclear whether the microbial communities associated with cognitive performance are functionally transferable and capable of influencing cognitive and molecular changes in recipient hosts. Experimental approaches such as fecal microbiota transplantation (FMT) provide an opportunity to investigate the causal contribution of donor-associated microbiota to host cognitive phenotypes. Mice share several physiological and immunological features with humans, and the microbiota delivered from humans can be engrafted in recipient mice.^25^

Here, we investigated the role of the gut microbiota in cognitive performance in older adults and adult mice. We hypothesized that different microbial taxa and functions are associated with cognitive performance and play a role in modulating brain function. We assessed SDMT scores and fecal shotgun metagenomics in older adults to identify microbial species and functions associated with cognitive performance and impairment. Next, we evaluated changes in cognitive performance in recipient mice following FMT from human donors with differing SDMT scores, as measured by the Morris water maze and neuronal density. Finally, we explored the mechanisms underlying microbiota-driven effects on cognitive performance by integrating human and mouse microbial functionality with hippocampal metabolism and cortical gene expression in mice.

## Methods

### Human study

#### Aging imageomics cohort

The Aging Imageomics study^26^ (*n* = 1030) is an observational study conducted in Girona (Northeast Catalonia, Spain), incorporating participants from two independent cohorts: The Maturity and Satisfactory Ageing in Girona study (MESGI50) and the Improving interMediAte RisK management study (MARK).^23^ Data were collected at Dr Josep Trueta University Hospital between November 14, 2017, and June 19, 2019, with ethical approval granted by the hospital's ethics committee. Samples and data from participants included in this study were provided by the IDIBGI Horizontal Aging Program and the IDIBGI Biobank from the Aging Imageomics Study. They were processed following standard operating procedures with the appropriate approval of the Ethics and Scientific Committees (CEIm Code 2017.146). The Aging Imageomics Study is an observational study including participants from the province of Girona (Northeast Catalonia, Spain). A detailed description of the cohort has been published previously.^26^ Briefly, participants were invited by telephone to participate in the Aging Imageomics Study. During this standardized telephone call, potential candidates were provided with information about the study and invited to request further details if desired. Participants who agreed to take part were asked to choose a date and time to complete the enrollment procedures. Candidates were scheduled to attend two visits. The first visit comprised three parts. First, candidates were informed in detail about the study aims and characteristics. Second, candidates who provided written informed consent were assigned a personal identification code. Third, a research assistant provided participants with a collection kit and detailed step-by-step instructions for collecting and transporting morning urine and stool samples, which were to be submitted on a second visit scheduled 15 d later. Reutilization of samples and data was approved by the CEIm Girona under approval code 2023.239.

Eligibility criteria included age ≥ 50 y, residing in the community, no history of infection in the last 15 d, no contraindications to magnetic resonance imaging (MRI), and consent to be informed of potential incidental findings. Extensive details of the study can be viewed elsewhere.^26^ Cohort population characteristics are detailed in the Supplementary Information (Table S1).

#### Symbol digit test

Participants were required to substitute a number for randomized presentations of geometric figures. Participants were presented with a key that pairs digits 1–9 with symbols and were asked to write down the corresponding numbers for the presented symbols. For the first 10 items, subjects received guidance to complete the task. They were then given 90 seconds to complete as many pairings as possible; higher scores represent better performance.

#### Shotgun fecal metagenomics of Imageomics cohort

Total DNA was extracted from frozen human stool samples using the PowerSoil DNA Isolation Kit (MO BIO Laboratories, Carlsbad, California, USA). Metagenomic libraries were prepared using the Illumina DNA Prep Kit (Illumina) with 400–500 ng of input DNA. Library quality was assessed using the TapeStation High Sensitivity DNA Kit (Agilent Technologies) and quantified with the Qubit fluorometer (Invitrogen). Libraries were pooled in equimolar concentrations and sequenced on the Illumina NextSeq 2000 platform, generating paired-end 150 bp reads. Raw reads were initially filtered using an in-house Python script to retain only those with a quality value (QV) > 30. Further preprocessing, including adapter trimming and quality filtering, was performed using fastp.^27^ To remove host-derived sequences, reads were mapped to the human genome (GRCh38.p13) using Bowtie2,^28^ and only unmapped reads were retained for downstream analysis. Metagenomic assembly and annotation were carried out using SqueezeMeta v1.3.1^29^ in co-assembly mode, which pools all samples into a single assembly. Contigs were assembled with MEGAHIT,^30^ and reads were mapped back to contigs using Bowtie2. Prodigal v2.6.3^31^ was employed for open reading frame prediction and Predicted protein sequences were searched against the GenBank non-redundant database using DIAMOND^32^ and taxonomic assignments were inferred. Functional annotations, including Kyoto Encyclopedia of Genes and Genomes (KEGG) subcategories and pathway-level assignments, were performed using HMMER against the 2016 KEGG database.^33^ Annotation refinement, read quantification, and abundance matrix construction were conducted in R software (v4.2.2).^34^

### Preclinical mouse study

#### Animals

Six-month-old male C57BL/6 mice (*n* = 66) were obtained from Janvier Labs (Saint Berthevin Cedex, France) and used in accordance with European guidelines following the approval of The Animal Ethics Committee of the Technological Unit of Nutrition and Health of EURECAT (Reus, Spain) and the Generalitat de Catalunya (protocol code 10886). The study complied with the EU Directive 2010/63/EU for animal experiments, the “Principles of Laboratory Animal Care” and the ARRIVE guidelines.^35^ Sample size was determined based on previous literature, while considering the feasibility of the number of animals required for the planned experimental procedures. Throughout the study at the EURECAT animal facility, mice were kept in groups of three per cage under a 12:12 h light–dark cycle at 22 °C. Mice had ad libitum access to water and standard chow (Teklad Global 14% Protein Rodent Diet 2014, Harlan, Barcelona, Spain). Body weight and food consumption were logged once a week. Mice were randomized into groups while considering body weight to ensure a balanced weight distribution. All experimental procedures were randomized across groups to minimize potential order effects and were performed by trained personnel blinded to donor SDMT scores. Mice were monitored during and after each experimental procedure to ensure animal welfare and to identify any signs of discomfort or distress.

#### Fecal microbiota transplantation

Thirteen male donors were selected to perform an FMT experiment on mouse recipients. SDMT scores among donors ranged from 13 to 77, with an average of 40. Given the limited number of donors selected for the FMT experiment, only male donors were included to eliminate donor sex as a source of variation in microbiome composition. Male recipient mice were subsequently selected to maintain a sex-matched donor–recipient experimental design. Donors were classified as high or low SDMT based on the median SDMT scores. Donor characteristics are detailed (Table S2). Human stool samples from the thirteen donors were handled on ice under a nitrogen atmosphere to maintain microbial viability. Each sample was diluted in sterile 0.9% NaCl, thoroughly homogenized, and spun at 179 × *g* for 5 min at 4 °C. The resulting supernatant was adjusted to a final concentration of 130 mg feces mL⁻^1^ in NaCl containing 10% glycerol. Aliquots of 0.6 mL were then flash-frozen in liquid nitrogen and stored at −80 °C until transplantation.

Fecal samples from donors with high or low SDMT were transplanted into adult, male 7.5-month-old C57BL/6 mice. Each donor sample was transplanted into three individual mice (*n* = 3 per donor). Healthy control mice (*n* = 10) that received sham gavages, and antibiotic control mice (*n* = 10) that received antibiotics and sham gavages were also included in the study.

Prior to FMT, an antibiotic cocktail containing a dose of 100 mg/kg for vancomycin and 200 mg/kg for neomycin, gentamicin, and metronidazole was administered by oral gavage once daily for 9 consecutive days to deplete the existing gut microbiota. Six molar hydrochloric acid (HCl) was added to the cocktail at 3% (v/v) of the total sample volume to improve solubility. Additionally, ampicillin was added to the autoclaved drinking water at a concentration of 1 g/L throughout the antibiotic treatment. A 72-h recovery period was allowed prior to fecal transplantation.

Omeprazole was administered to recipient mice at 40 mg/kg body weight by oral gavage 4 h before FMT to block proton pump activity and protect the microbiota from high acidity. One hundred and fifty microliters of FMT at a concentration of 130 mg/ml was administered by oral gavage for 3 consecutive days in week one, followed by twice a week for 4 weeks to establish the microbiota prior to behavior tests, following guidelines previously described.^25^ FMT continued twice a week until sacrifice at 8 weeks.

#### Sample collection and sacrifice

Fecal samples were collected from the cecum following 8 weeks of FMT. All samples were stored at −80 °C until further processing. In week 8 of FMT, mice underwent a 6-hour diurnal fast, prior to sacrifice by decapitation. The cecum, hippocampus, and cortex from the right hemisphere were collected, weighed, snap-frozen in liquid nitrogen, and stored at –80 °C until further analysis. In addition, the left hemisphere of the brain was collected and fixed in buffered formalin (4% formaldehyde, 4 g/L NaH_2_PO_4_, 6.5 g/L Na_2_HPO_4_; pH 6.8) for histological analyzes.

#### Cognitive test

At week 4 after starting the FMT procedure, mice underwent the Morris water maze (MWM) for spatial learning and working memory using a modified version outlined previously.^36^ Mice were trained over 4 d with 4 trials per day with search times recorded to locate the submerged platform. Each test day, mouse navigation was evaluated using ANY-MAZE software. Time to platform (s) was recorded manually with a stopwatch, and path efficiency and corrected integrated path length (CIPL) were recorded using ANY-MAZE software. CIPL is the difference between the sum of the sampled distances from the submerged platform and the shortest possible sum if the mouse had swum directly to the hidden platform at its mean speed.

#### Shotgun fecal metagenomics of mouse recipients

DNA extraction was performed using the Fast DNA Stool Mini Kit (Qiagen, Hilden, Germany) according to the manufacturer's instructions, adapting reagent volumes according to initial volumes of fecal material, which ranged between 20 and 50 mg. The procedure used efficient lysis and silica membrane-based purification to maximize the removal of PCR inhibitors. DNA was eluted in 50 μL of nuclease-free water and quantified using the Qubit 4.0 fluorometer and the Qubit 1× dsDNA High Sensitivity Assay Kit (Thermo Fisher). One hundred to five hundred nanograms of DNA was used for library preparation with the Illumina DNA Prep with Tagmentation kit. Dual indexes (IDT DNA Dual Indexes) were ligated according to the manufacturer's instructions, with the number of PCR cycles adjusted for samples with input DNA below the recommended value. Libraries were quantified using the Qubit 4.0 fluorometer with the High Sensitivity DNA Kit, and size distribution was determined by microfluidic electrophoresis using the Agilent TapeStation system, with the Agilent High Sensitivity DNA Kit. Libraries failing to meet the quality thresholds of ≥750 pM concentration and 400–600 bp size were excluded. Qualified libraries were pooled equimolarly at 750 pM, loaded onto Illumina sequencing reagents (P2 and/or P3, 300 cycles) and clustered using a compatible flow cell. Paired-end sequencing (2 × 150 bp) was performed on the Illumina NextSeq 2000, with resulting raw data demultiplexed and converted to FASTQ format. Taxonomic assignment was performed as in humans using the mouse genome (GCF_000001635.27).

#### CIL LC-MS metabolomics

An assay to detect metabolites was performed in mouse hippocampal tissue samples using chemical isotope labeling (CIL) liquid chromatography mass spectrometry (LC–MS) with the Dansyl-labeling kit or DmPA-labeling kit. Samples were randomized to minimize technical variation. Metabolites were extracted from hippocampal tissue prior to making aliquots. Samples were transferred to steel tubes containing two 6.35 mm diameter steel beads, weighed, and homogenized using bead-beating at 6 m/s for 15 s. Metabolites were extracted by adding 500 μL of cooled MeOH/water (4:1), followed by further homogenization at 4 m/s for 10 s. Homogenates were incubated at −20 °C for 10 min and centrifuged at 15,000 × g for 10 min. The supernatant was transferred to a fresh vial, dried, and resuspended in 50 μL of LC–MS-grade water. Samples were adjusted to a total metabolite concentration of 82 mM following metabolite determination via the NovaMT Sample Normalization Kit.

Samples were vortexed and centrifuged at 15,000 × g for 5 min, with supernatants aliquoted for amine/phenol labeling and carboxyl labeling. Pooled reference samples were generated by combining equal volumes from each individual sample.

For amine/phenol labeling, each aliquot was mixed with 12.5 μL of buffer Reagent A and 37.5 μL of Reagent B, containing either ^12^C₂-label (individual and pooled samples) or ^13^C₂-label (pooled samples), and incubated at 40 °C for 45 min. Reactions were quenched with 7.5 μL of Reagent C and incubated at 40 °C for 10 min, followed by the addition of 30  μL of pH-regulating Reagent D.

For carboxyl labeling, each aliquot was mixed with 10 μL of catalyzing Reagent A and 25 μL of Reagent B (^12^C_2_-label for individual and pooled samples or ^13^C_2_-label for pooled samples) and incubated at 80 °C for 60 min. Reactions were quenched by adding 40 μL of Reagent C and incubated at 80 °C for 60 min.

Equal volumes of ^12^C_2_-labeled individual samples were combined with ^13^C₂-labeled reference samples and analyzed by LC–MS. A quality control (QC) sample was prepared by mixing equal volumes of ^12^C_2_- and ^13^C_2_-labeled pooled samples.

LC–MS analysis, data processing, and metabolite identification were performed as described previously,^17^ with QC samples injected every 10 runs. 879 metabolites were identified with >90% confidence (Table S3).

#### Cortical RNA sequencing

Total RNA was extracted from medial prefrontal cortex (mPFC) tissue, and RNA quality was assessed using the Agilent Bioanalyzer 2100, with RNA integrity numbers > 7. Libraries were prepared from 500 ng^–1^ μg of total RNA using the TruSeq Stranded mRNA kit (Illumina) according to the manufacturer's instructions. Library quality and quantity were evaluated by TapeStation (Agilent) and KAPA qPCR quantification. Sequencing was performed on an Illumina platform (HiSeq 2500 or NovaSeq) with paired-end reads (2 × 50 or 2 × 100 bp). Reads were aligned to the GRCm38.p6 mouse reference genome (Gencode release 17) using STAR (v2.5.3a),^37^ with gene-level counts obtained using FeatureCounts (Subread v1.5.1).^38^

#### Brain histological analysis

Left cerebral hemispheres were fixed in a 4% diluted formaldehyde for >48 h, coronally sectioned at 3–4 mm and underwent successive dehydration through graded ethanol solutions (70%, 96% and 100%) followed by xylol (Dimethyl benzene) treatment and paraffin infiltration-immersion at 65 °C (ASP6025, Leica Biosystems). Three-micrometer thick sections (Galileo, Diapath) were obtained using a training paraffin block (EC 350-1, Myreva) and were placed on slides (JP Selecta Paraffin Bath) for automated hematoxylin – eosin staining (Gemini AS. Epredia). For Nissl staining, tissue sections were rinsed twice with 0.01 M PBS for 5–10 min, stained in a pre-warmed 0.5% cresyl violet solution for 10 min, washed with distilled water, and then dehydrated through a graded ethanol series (70%, 95%, and 100%). Tissue slices were cleared in xylene for 5 min, and glass coverslips were applied using a mounting medium.

Neurone density was quantified by microscopy using a Leica DM750 microscope, and the image was taken with Aperio ImageScope Digital Pathology Scanner software (Software Leica Application V12.4.6.5003 Aperio AT2, Leica Biosystems) by two examiners blinded from the experimental procedure of the preclinical trial.

### Statistical analysis

Statistical analyzes were performed using R (V4.5.0.). Normality and homogeneity of variances were assessed visually and with the Shapiro‒Wilk test for normality. To account for multiple comparisons, SGoF correction was applied to the high-dimensional omics analyzes involving large numbers of simultaneous tests, while the Benjamini–Hochberg (BH) FDR procedure was applied to smaller, targeted sets of comparisons, including MWM cognitive analyzes and histological analyzes. Unless otherwise stated, all recipient mice (*n* = 39) were included in the analyzes. Where fewer mice were included, this was due to sample unavailability resulting from technical or experimental limitations.

#### Metagenomics analysis

For functional metagenomics, briefly, functional orthologues were filtered so that only those with at least 10 reads present in 20% of samples were included in the analysis. Using the “ANCOM-BC” R package, differential abundance analyzes were performed to identify taxa and KEGG orthologues associated with human SDMT scores, controlling for age, sex, BMI, and years of education.^39^ *P* values were adjusted for multiple comparisons using the Sequential Goodness of Fit (SGoF) R package.^40^ Unlike false discovery rate (FDR) methods, which typically lose statistical power as the number of comparisons increases, SGoF methods gain power with increasing numbers of tests. SGoF has been shown to outperform FDR approaches when there are high numbers of tests and low sample sizes, making it particularly well suited to large omics datasets. Significance was set at *P*adj < 0.05 for Imageomics cohort and *P*adj < 0.1 in mouse recipients. Given the large number of participants in the human cohort, an adjusted significance threshold of *P*adj < 0.05 was applied to provide stringent control of false-positive findings. In contrast, the mouse experiments (*n* = 38 mice) included a limited number of recipients and were designed as exploratory mechanistic analyzes; therefore, a threshold of *P*adj < 0.10 was applied to increase sensitivity to potentially biologically relevant signals while maintaining control for multiple testing. Findings of the mouse analyzes were therefore interpreted as exploratory and require confirmation in larger independent experiments. Over-representation and gene sequence enrichment analyzes were carried out using the package “clusterProfiler”^41^ to determine differentially expressed functional orthologues as expressed by Kyoto Encyclopedia of Genes and Genomes (KEGG) orthology in differing cognitive groups.^33^ Engraftment analyzes were restricted to available samples and to recipient mice for which the corresponding human donor sample was analyzed, comprising 12 human donors, 36 FMT recipient mice, and 9 control mice. Zero counts were replaced using the zCompositions R package, followed by centered log-ratio (CLR) transformation and principal component analysis (PCA) using the prcomp function in R.^42^ Species overlap between human donors, FMT recipient mice, and control mice was visualized using a Venn diagram. Engraftment rates were calculated by matching each recipient mouse to its corresponding human donor and determining the percentage of donor microbial species detected in the matched recipient.

#### Metabolomics analysis

Mouse hippocampal LC‒MS data (*n* = 38 mice) were normalized using a probabilistic quotient normalization,^43^ which corrects for differences in sample dilution and overall metabolite abundance between samples, and analyzed initially by applying a linear model to the data using the R package “limma”.^44^ Metabolites associated with cognition scores were identified using robust linear regression models adjusted for age, BMI, sex, and years of education. *P* values were adjusted for multiple comparisons using the SGoF, to determine differentially expressed metabolites, with significance set at *p*adj < 0.1. The R packages “MicrobiomeProfiler” and “clusterProfiler” were implemented to perform KEGG pathway enrichment analysis.^41^ Metabolites were mapped using the KEGG database.

#### RNA sequencing analysis

The limma (Linear Models for Microarray Data) R package was used to determine differentially expressed gene counts of the mouse mPFC (*n* = 37 mice) associated with donor SDMT scores.^44^ Low-abundance genes were first filtered so only genes with > 10 counts in 20% of samples were kept for analysis. Using the “edgeR” package, data were normalized using the trimmed mean of M-value.^45^ Using the “voom” function in the limma package, normalized counts were then converted to log_2_ count per million with associated precision weights to account for differences in precision between different observations, controlling for the covariates of age, BMI, and years of education. Using the “lmFit” function, a robust linear regression model controlling for these covariates was fitted to the data. Using the “eBayes” function, an empirical Bayes method was applied to improve variance estimation by borrowing information across genes. *P* values were adjusted for multiple comparisons using the SGoF procedure, with an adjusted p-value threshold of <0.10. Over-representation analysis was performed using KEGG databases to determine functional roles of differentially detected genes. Pathway enrichment significance was assessed using q-values, with pathways considered significant at <0.05.

#### MWM analysis

MWM outcomes (*n* = 39 mice; 3 mice per donor) were analyzed using linear mixed-effects models in R with the “nlme” package, incorporating an interaction between test day and donor SDMT group, with random intercepts for human donor and individual mouse ID nested within donor. For a sensitivity analysis, models were performed both unadjusted and adjusted for donor age, BMI, and years of education. Donor SDMT scores were analyzed both as a group factor and a continuous predictor. Models were fitted using restricted maximum likelihood (REML). Statistical significance of fixed effects was assessed using Type III ANOVA implemented in the “car” R package. Normality and homoscedasticity assumptions were assessed through visual inspection of residual and quantile–quantile (QQ) plots. Both path efficiency and CIPL data were square-root transformed to obtain a normal distribution. Post hoc comparisons of estimated marginal means were performed using the “emmeans” package, with BH correction, to compare the high- and low-SDMT groups at each test day. *p < 0.05.

#### Histology

Neurone density was quantified in triplicate for each sample, and mean values were calculated prior to analysis. To account for multiple recipient mice (*n* = 3 per donor), mouse neuronal density values were averaged within each donor. Spearman correlation analyzes (*n* = 13 donors) were performed between SDMT scores and neurone density, with *p*-values adjusted for multiple comparisons using the BH FDR.

## Results

### Enrichment of species and microbial functions is associated with SDMT score in human participants of the Imageomics cohort

To identify compositional and functional microbiota signatures associated with cognitive function in older individuals, we analyzed the associations between SDMT scores and the microbiota in the ageing Imageomics cohort (*n* = 867; Figure 1a; Table S4). Twelve microbiota species were found to be associated with higher SDMT scores after controlling for age, sex, BMI, and years of education. These included members of the Muribaculaceae family such as *Muribaculum caecicola*, Muribaculaceae bacterium Isolate-002 (NCI), *Duncaniella freteri,* unclassified Muribaculaceae, Muribaculaceae bacterium, and Muribaculaceae bacterium Isolate-013 (NCI), along with members of other families such as *Barnesiella* sp. WM24 and *Ruminococcus* sp. J1101004 170508 H5 (Figure 1b). Several species were also associated with lower SDMT scores, including *Eubacterium* sp. AF22-8LB, Unclassified *Raoultella*, *Eubacterium* sp. AM28-29, and *Holdemanella* sp. MSK.7.32.

**Figure 1.**
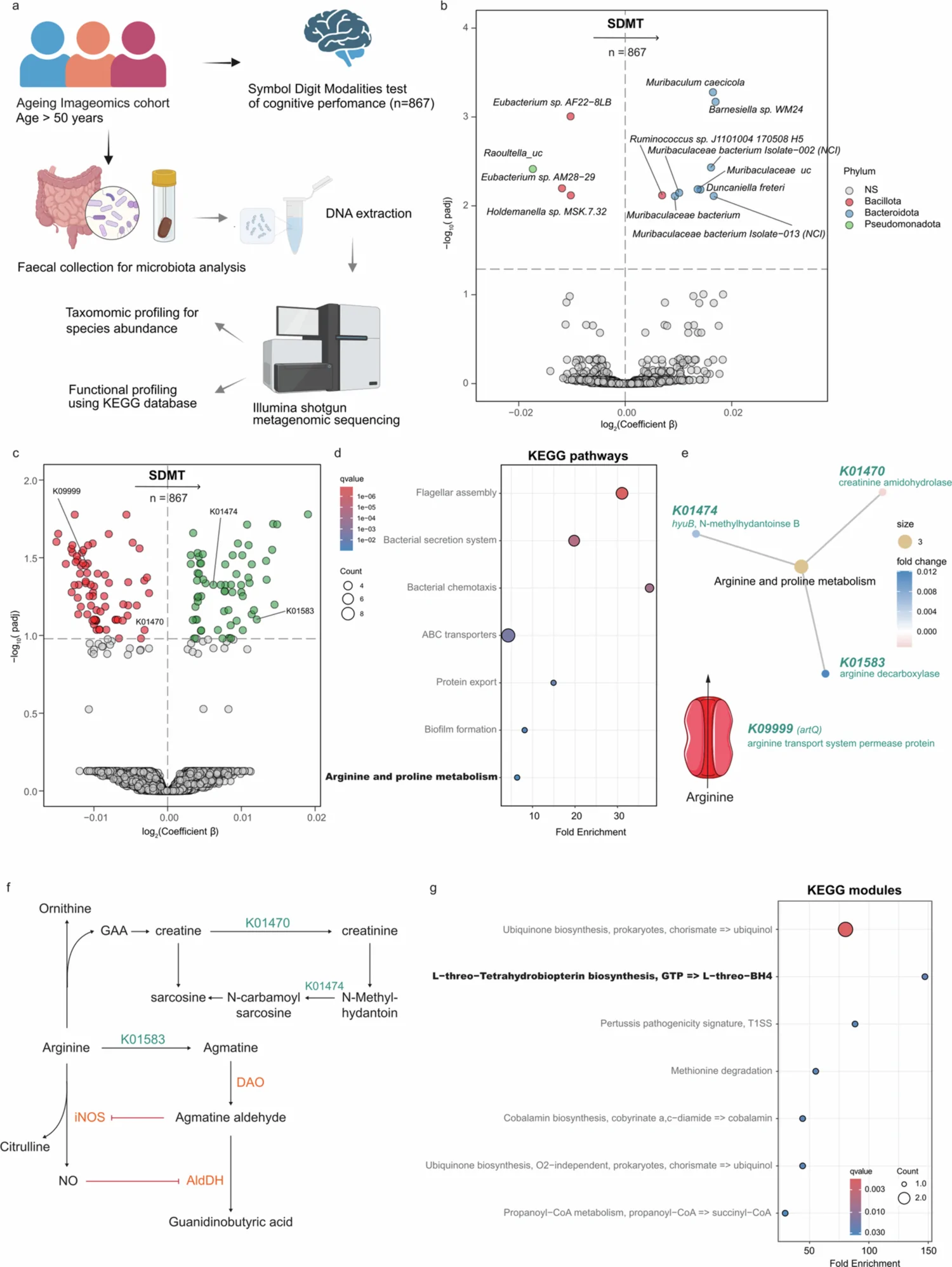
(a) Schematic of Imageomics cohort analysis. (b) Human imageomics cohort Volcano plots showing up and down-regulation associated with SDMT after correcting for donor age, BMI, and years of education. Over representation analysis. Plots showing which microbiota are enriched and downregulated in association to human SDMT score. (c) Volcano plots of differential microbial molecular functions associated with SDMT in the human Aging Imageomics cohort (Significant associated genes were identified using the ANCOM-BC from shotgun metagenomics data, controlling for age, sex, BMI, and years of education. The log2 fold change of the association with a unit change in the ANCOM-BC-transformed variable values and the log10 *p*-values adjusted for multiple comparisons using a sequential goodness-of-fit were plotted for each taxon. Significance was set at *p*adj < 0.05. Significantly different genes are colored in green (upregulated) or red (downregulated). (d) Bar plots of the over-representation KEGG pathway analyzes (q-value < 0.1) mapping the KEGG orthologues significantly associated with SDMT in the human Aging Imageomics cohort. (e) Corresponding C-net plot of the arginine and proline pathway and ABC transporters showing significantly altered KEGG orthologues (f) Schematic representation of microbial metabolic pathways associated with SDMT scores, including creatinine/sarcosine metabolism and corresponding KEGG orthologues. (g) Bar plots of the over-representation functional KEGG modules (q-value < 0.1) significantly associated with SDMT in the human Aging Imageomics cohort.

Next, a functional analysis was performed by mapping reads to KEGG orthologues using ANCOM-BC, controlling for age, sex, BMI, and years of education (Figure 1c; Table S5). Results from an overrepresentation analysis revealed that several microbial pathways were significantly associated with SDMT scores (Figure 1d; Table S6), including arginine and proline metabolism and ABC transporters. The arginine decarboxylase (K01583), which converts arginine to agmatine, and *N*-methylhydantoinase (K01474), which converts *N*-methyl-hydantoin to *N*-carbamoyl sarcosine, were positively associated with SDMT (Figure 1c and e). Conversely, creatine amidohydrolase (K01470) was negatively associated with SDMT (Figure 1c, e, and f). Among the ABC transporters, the arginine transport system permease (K09999) was negatively associated with SDMT (Figure 1c and e). A schematic diagram illustrates these metabolic pathways and their relevant KEGG orthologues (Figure 1f). Furthermore, a KEGG module over-representation analysis revealed a significant enrichment of the L-threo-tetrahydrobiopterin (L-threo-BH4) biosynthesis module associated with SDMT (Figure 1g; Table S7). Tetrahydrobiopterin (BH4) is an essential enzymatic cofactor for aromatic amino acid hydroxylases involved in tyrosine and tryptophan metabolism and for enzymes that utilize L-arginine as a substrate.^46^

### FMT from donors with high SDMT significantly improved learning of recipient mice along with increasing neurone density

Microbiota of thirteen male donors with high and low SDMT scores were transferred to recipient mice. Fecal samples of each donor were transplanted into 3 recipient mice. To confirm the engraftment of human microbiota, we analyzed the recipient mouse microbiota using shotgun metagenomic sequencing. PCA of CLR-transformed metagenomic data showed a separation between the microbiota of FMT recipient and control mice (Supplementary Figure 1a). FMT recipient mice shared 753 species with human donors, corresponding to 81.8% species overlap, with 84 of these species absent in control mice (Supplementary Figure 1b). When recipient mice were matched to their respective human donors, the median species-level engraftment rate was 53.7% (IQR, 50.4%–57.7%) (Supplementary Figure 1c). Spatial learning and memory in recipient mice were assessed using the MWM over 4 d (Figure 2a). The microbiota of human donors with different SDMT scores had a significant effect on mouse MWM performance, with recipient mice receiving microbiota from donors with high SDMT scores performing better than those receiving microbiota from donors with low SDMT scores (Figure 2b).

**Figure 2.**
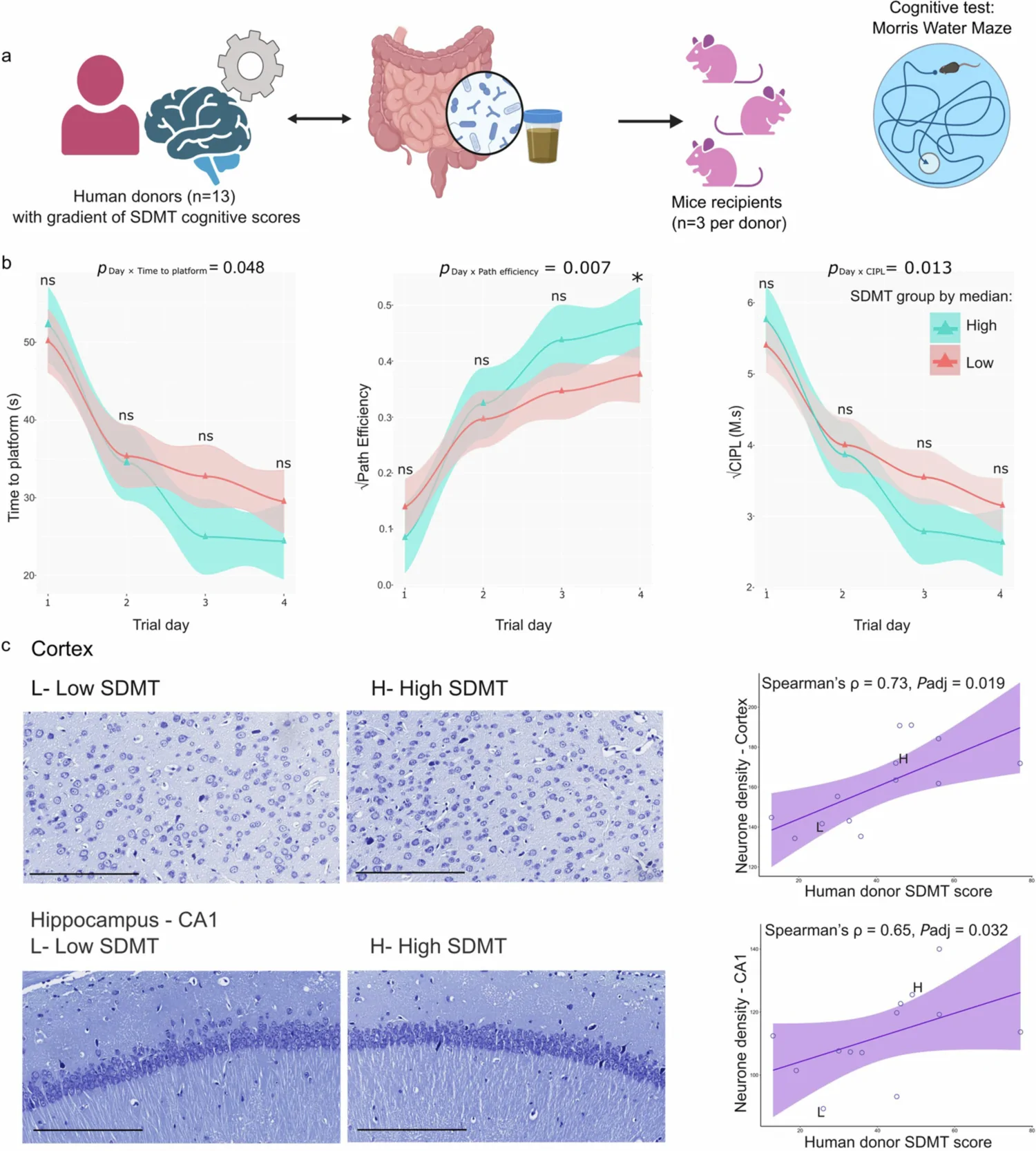
(a) Schematic of the study design. Fecal microbiota transplantation (FMT) was performed in mice using stool from human donors with high and low SDMT cognitive scores. Following the FMT period, mice underwent behavioral testing and omics profiling. Morris Water maze (MWM) tests showing: Time to platform; Path efficiency; Corrected Integrated Path Length (CIPL). (b) Data analyzed by stratifying recipient mice into high and low SDMT cognition groups based on the median SDMT score of the human cohort, using a mixed linear model with the interaction between test day and donor SDMT scores. Lines show model-estimated means (shaded 95% confidence intervals). Pairwise post-hoc are annotated *p < 0.05. (c) Representative Nissl staining of the cortex and CA1 region of the hippocampus in recipient mice, showing the relationship between human donor SDMT scores and neuronal density in recipient mice. Each point represents one human donor, with neuronal density calculated as the mean across recipient mice receiving FMT from that donor. Scale bar represents 200 μm. Corresponding Spearman correlations are displayed.

Using a linear mixed-effects model with the interaction of the SDMT group factor with time, a significant interaction was found for time to platform (*p* = 0.048), path efficiency (*p* = 0.007), and CIPL (*p* = 0.013) (Figure 2b; Table S8). These interactions remained significant after adjustment for donor BMI, education, and age, for time to platform (*p* = 0.048), path efficiency (*p* = 0.007), and CIPL (*p* = 0.013). This was evident on day 4, when recipient mice receiving microbiota from high-scoring SDMT donors exhibited greater path efficiency than those receiving microbiota from low-scoring SDMT donors (mean ± SD: 0.289 ± 0.096 vs 0.201 ± 0.142, respectively; *p* = 0.038; Table S9). Additionally, whenSDMT was analyzed as a continuous variable, mice receiving microbiota from donors with higher SDMT showed significantly improved learning when compared to recipient mice receiving microbiota with lower SDMT, as measured by time to platform (*p* = 0.027), path efficiency (*p* = 0.001) and CIPL (*p* = 0.010; Table S10).

Nissl staining revealed a positive association between donor SDMT scores and neuronal density in recipient mice, with neuronal density averaged across recipient mice colonized with microbiota from each human donor. This association was observed in the cortex (*ρ* = 0.73, *p* = 0.019) and in the Cornu Ammonis 1 (CA1) region of the hippocampus (*ρ* = 0.65, *p* = 0.032; Figure 2c; Table S11), indicating that mice colonized with microbiota from donors with higher SDMT cognitive scores exhibited greater neuronal density.

### Following FMT, *Muribaculum caecicola,* and *Duncaniella freteri* were more abundant in high-SDMT recipient mice, alongside coordinated changes in gut microbial functions

Analysis of the cecal microbiota composition of the recipient mice associated with the donor SDMT revealed that microbial species that were more abundant in the feces of humans with high SDMT, such as *M. caecicola* and *D. freteri,* were also more abundant in the cecal microbiota of recipient mice receiving microbiota from human donors with high SDMT (Figure 3a; Table S12).

**Figure 3.**
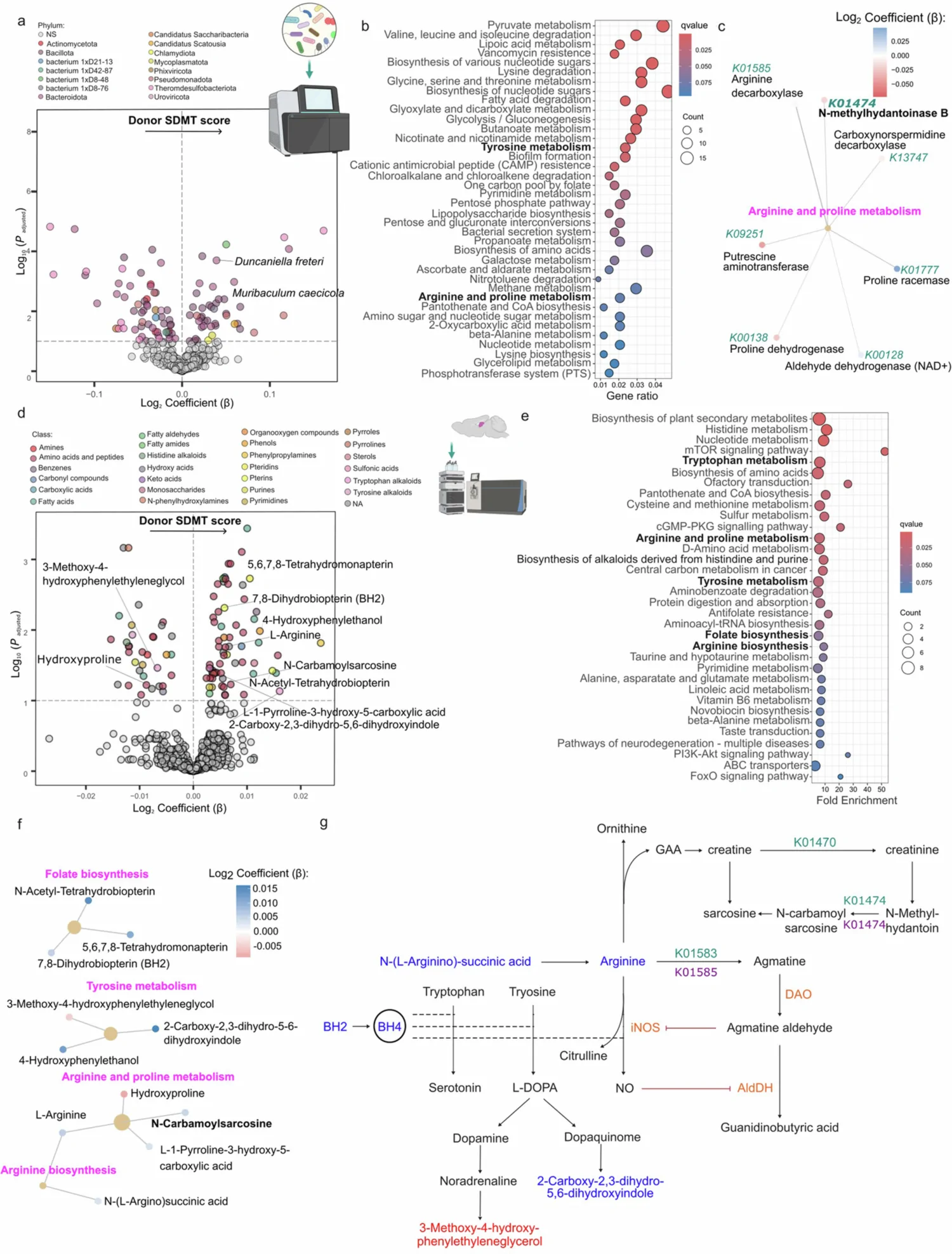
Metagenomics analysis of the microbiota and metabolomics of the hippocampus showing data associated with SDMT in the mouse recipients receiving FMT from the selected 13 human donors. All analyzes were controlled for age, BMI, and years of education. (a) Volcano plots of differentially abundant microbial species identified using the ANCOM-BC from shotgun metagenomics. The log2 fold change of the association with a unit change in the ANCOM-BC-transformed variable values and the log10 *p*-values adjusted for multiple comparisons using a sequential goodness-of-fit were plotted for each taxon. Significance was set at *P*adj < 0.1. Significantly different phyla are colored accordingly. (b) Bar plots of the over-representation KEGG pathway analyzes (q value < 0.1) mapping the KEGG orthologues significantly associated with donor SDMT scores. (c) C-net plot of differential microbial genes of the arginine and proline metabolic pathway following over-representation KEGG pathway analyzes (q value < 0.1) mapping the KEGG orthologues to determine overexpressed metabolic pathways. (d) Volcano plots of differential hippocampal metabolites identified applying a linear model to the data using the R package “limma” on metabolites identified by CIL-LC-MS. Significance was set at *p*adj < 0.1. Significantly different metabolite classes are colored accordingly. (e) Over-representation analysis using the metabolites associated with donor SDMT scores, identified with limma. Dots are colored according to the q-value. (f) C-net plot of differential metabolic pathways of the arginine biosynthesis, arginine and proline, tyrosine and folate metabolic pathways (q value < 0.1). (g) Schematic representation of metagenomic and metabolic pathways centered around the co-factor BH4, neurotransmitter synthesis and metabolic pathways associated with SDMT scores. For recipient mice, upregulated metabolites are colored in blue, while downregulated metabolites are shown in red. Differentially abundant KEGG orthologues are colored in green for humans and purple for recipient mice.

Functional analyzes using ANCOM-BC controlling for donor age, BMI, and years of education (Supplementary Figure 3a; Table S13), followed by KEGG pathway over-representation analyzes identified several pathways significantly associated with the donor SDMT scores (Figure 3b; Table S14). Consistent with the findings in humans, arginine, and proline metabolism were significantly associated with the donor SDMT scores. Notably, the arginine decarboxylase (K01585) and the *N*-methylhydantoinase B (K01474) were among the KEGG orthologues involved in arginine and proline metabolism (Figure 3b and c). Furthermore, the tyrosine pathway was also overrepresented in association with the donor SDMT scores (Figure 3b, Supplementary Figure 3b).

### Metabolites of the arginine and pterin pathways were enriched in the brains of mice receiving microbiota from high SDMT donors

Next, we performed global metabolic profiling by CIL LC-MS of the hippocampus of recipient mice and identified metabolites associated with the donor's SDMT scores using a robust regression controlling for the donor age, BMI, and years of education (Figure 3d; Table S15). A KEGG pathway overrepresentation analysis again highlighted enrichment of arginine and proline metabolism and arginine biosynthesis pathways associated with the SDMT scores (Figure 3e; Table S16). L-Arginine and its precursor *N*-(L-Arginino) succinic acid were both increased in the hippocampus of recipient mice receiving microbiota from donors with higher SDMT scores. Notably, in line with our metagenomic findings in humans and mice, *N*-carbamoyl sarcosine, the product of K01474, was among the most significant metabolites in arginine and proline metabolism (Figure 3f). In addition, the folate biosynthesis across the biopterin pathway was also enriched in association with the donor SDMT. Interestingly, the concentrations of 7,8-Dihydro-biopterin (BH_2_) N-acetyl-tetrahydrobiopterin and 5,6,7,8-Tetrahydromonapterin were increased in the hippocampus of recipient mice receiving microbiota from donors with higher SDMT. A schematic diagram details these metabolic pathways (Figure 3g).

### Donor SDMT scores were associated with altered dopaminergic signaling gene expression in recipient mouse brains

To determine whether FMT microbiota differences translate into transcriptional changes in the brain, RNA sequencing (RNA-seq) was performed on the cortex of recipient mice (Figure 4a; Table S17). Several neurotransmitter signaling pathways were enriched across different SDMT scores, including neuroactive ligand-receptor interaction, neuroactive ligand signaling, and dopaminergic signaling (Figure 4b and c, Supplementary Figure 4; Table S18). In agreement with reduced catecholamine biosynthesis of hippocampal metabolites, the gene Tyrosine hydroxylase (*Th*) was downregulated in the cortex of high SDMT mouse recipients (Figure 4a and c). BH_4_ is the cofactor for TH, which catalyzes catecholamine biosynthesis by the conversion of tyrosine to L-DOPA.^47^ Amongst other genes of the dopaminergic synapse, solute carrier family 18 member A2 (Slc18a2) was also reduced in these mice; this gene is responsible for the transport of dopamine into synaptic vesicles. Furthermore, the solute carrier family 6 member 3 (Slc6a3), which encodes the transporter responsible for dopamine reuptake into the presynaptic neurone, was also reduced in these mice. The dopamine receptors, dopamine receptor D1 (Drd1), dopamine receptor D2 (Drd2), and dopamine receptor D5 (Drd5), however, were all enriched in high SDMT recipient mice. Drd1 and Drd5 are responsible for the production of cyclic adenosine monophosphate (cAMP), and through protein phosphatase 1 regulatory inhibitor subunit 1B (Ppp1r1b), another enriched gene in these mice, synaptic plasticity is modulated. Drd2 counteracts Drd1/Drd5 signaling, suppressing cAMP-dependent signaling. A schematic of the dopaminergic synapse with relevant genes is displayed (Figure 4d). Additionally, G protein subunit gamma 4 (Gng4) was also found to be downregulated and is the last of the differentially expressed genes of the dopaminergic synapse. Gng4 is a *γ* subunit of heterotrimeric G proteins involved in Gi/o signaling, signaling which inhibits adenylyl cyclase and reduces cAMP levels.^48^ Together, these enrichments suggest greater dopaminergic modulation and balanced signaling in recipient mice receiving microbiota from high-SDMT donors.

**Figure 4.**
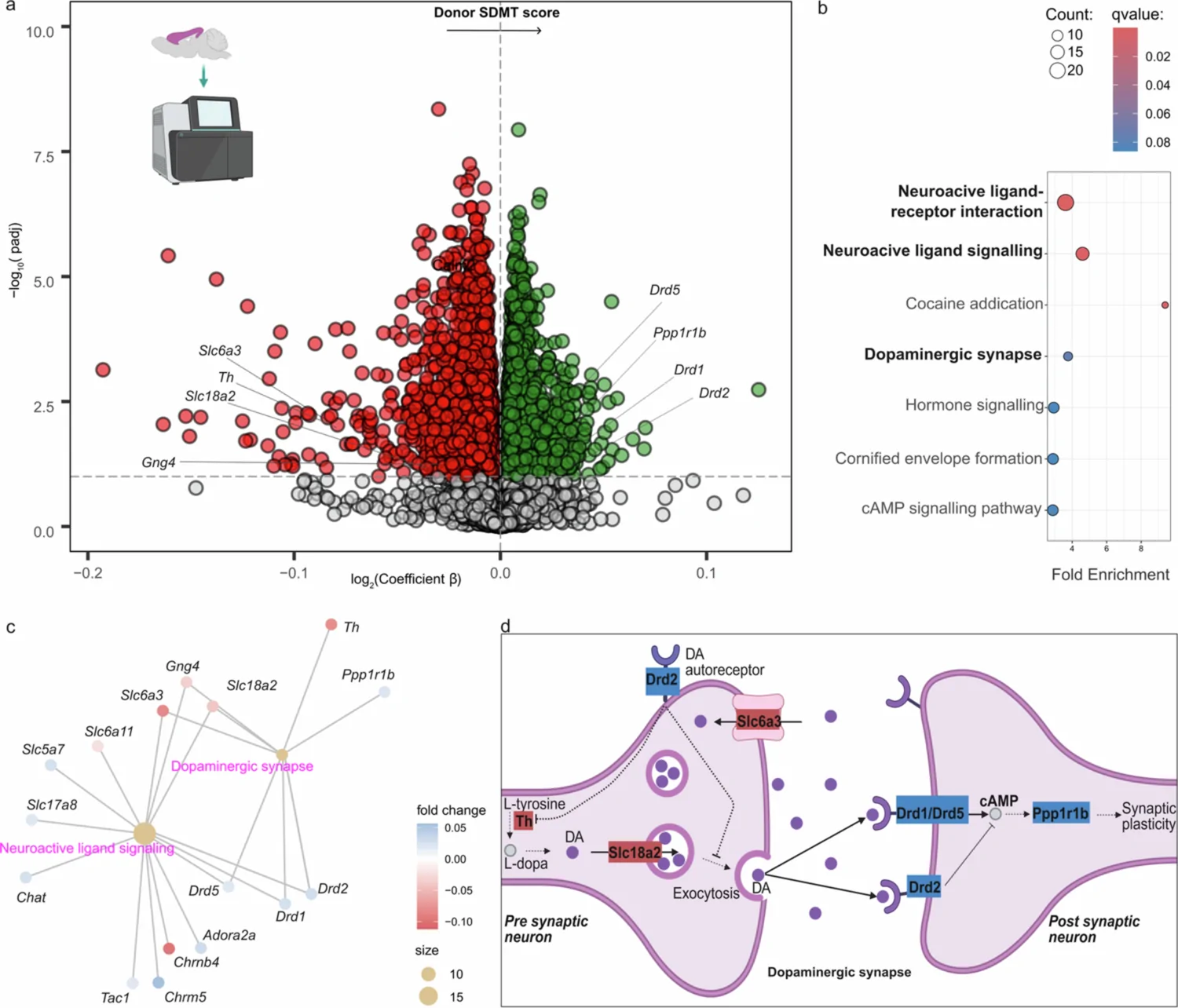
RNA-seq of the cortex in recipient mice controlled for age, BMI, and years of education. (a) Volcano plots of mPFC transcripts associated with donor SDMT following limma-voom analysis and correction for multiple comparisons. Genes of the dopaminergic synapse are labeled. (b) Bar plots of the over-representation RNA-seq pathway analyzes (q-value < 0.1) for genes significantly associated with donor SDMT. (c) Corresponding C-net plot of overexpressed pathways and genes corresponding to the Dopaminergic synapse and neuroactive ligand. (d) Schematic representation of gene alterations in the dopaminergic synapse where genes enriched are shown in blue and those downregulated are shown in red.

## Discussion

The role of the gut microbiota in ageing and health has become a hotspot for research in recent years.^49^ ^,^ ^50^ The need to understand the role of the microbiota in ageing is increasingly important as the ageing population grows worldwide. Much is known about how microbiota communicate with the brain via the gut–brain axis; however, less is known about the mechanistic properties of the microbiota that influence cognitive capabilities during ageing. In this study, we found compositional and functional differences in the human microbiota associated with varying cognitive abilities, as measured by the SDMT cognitive test, in the ageing Imageomics cohort. By combining human microbiome analyzes with a mouse FMT study, and performing integrative multi-omics analyzes including fecal metagenomics, hippocampal metabolomics, and mPFC RNA-seq, we defined functional properties of the microbiota that influence cognitive function in aged individuals. We also elucidated how these microbiota-driven metabolic pathways may improve cognitive function in aged individuals. Together, these findings support a model in which microbiota-associated metabolic remodeling contributes to cognitive performance during ageing.

We have demonstrated that microbiota associated with cognitive performance in older adults influence cognitive outcomes in recipient mice. FMT from human donors with higher SDMT cognitive scores improved spatial learning in mouse recipients compared with FMT from donors with lower SDMT scores. Previous studies have shown that young mice's cognitive function was impaired by FMT from aged mice.^51^ ^,^ ^52^ Conversely, microbiota from young mice can reverse age-associated cognitive decline in older mice.^53^ Our findings build upon previous studies by demonstrating that microbiota associated with cognitive performance in humans can influence cognitive performance, neuronal density, and host metabolic pathways in recipient mice.

Mouse recipients receiving microbiota from donors with higher SDMT scores showed increased neuronal density in both the CA1 region of the hippocampus and the cortex. The gut microbiota has been shown to influence brain structure and function, with FMT altering expression of hippocampal synaptic plasticity in recipient mice, and neurotransmission proteins and microglial cells of the hippocampal fimbria acquiring an ageing-like phenotype.^51^ FMT from aged to young mice has also been shown to result in microglia-mediated synaptic loss and damaged neurones partially due to a deficiency in *Bifidobacterium pseudolongum* and the metabolite indoleacetic acid.^52^ FMT from humans with differing bacteriophage populations into recipient mice influenced mice's cognitive function and expression of mPFC genes, while bacteriophage supplementation in *Drosophila* influenced expression of genes involved in synaptic plasticity, neuronal development and memory.^24^ The observed increases in neuronal density in the hippocampus and cortex further suggest that microbially derived metabolites may contribute to neuroplasticity or neuronal maintenance during ageing.

Mouse recipients receiving microbiota from donors with higher SDMT scores revealed an enrichment of *M. caecicola* and *D. freteri* in their fecal samples and an overrepresentation of arginine and proline metabolism when analyzing their microbiota. Notably, these taxa and functions were also upregulated in high SDMT-scoring humans. Muribaculaceae have been proposed as a potential probiotic due to their ability to metabolize plant foods, mucin glycans, and dietary fiber and their ability to produce short-chain fatty acids, along with their cross-feeding relationships with the probiotic genera *Lactobacillus* and *Bifidobacterium.* ^54^ Through functional analysis, arginine and proline metabolism was identified as significantly altered during ageing when comparing aged and young mice.^55^ Members of the Muribaculaceae family are known to contribute to amino acid metabolism; however, further research is required to determine their specific relationship with arginine and proline metabolic pathways.^54^ Advances in omics technologies, including metagenomics, metabolomics, and transcriptomics, have allowed detailed investigation of microbial functionality, driving a shift from microbial composition to microbial functionality.^56^ Increasing evidence indicates microbial-derived functions rather than taxonomic composition are more reflective of age-related phenotypes. Due to substantial functional redundancy, multiple taxa can carry out the same functions within the gut microbiome, resulting in shared microbial pathways amongst individuals, whereas microbial taxa show greater interindividual variability.^57^ Together, these findings suggest that microbial taxa and metabolic pathways associated with higher SDMT scores are conserved across humans and recipient mice and may represent microbiome signatures linked to healthy cognitive ageing.

Arginine, proline, and tyrosine metabolism were among the microbial functional pathways enriched in the microbiota of recipient mice and were also reflected in metabolic changes within the hippocampus. Notably, L-arginine levels were elevated in the hippocampus of mice receiving microbiota from high-SDMT donors. L-arginine serves as the primary substrate for nitric oxide synthase (NOS), which catalyzes the production of nitric oxide (NO), a signaling molecule involved in synaptic plasticity and learning processes within the brain.^55^ ^,^ ^58^ ^,^ ^59^ Increased L-arginine availability has also been associated with improved cognitive function in older adults.^60^ Intravenous infusion of L-arginine has been shown to induce vasodilation and reduce blood pressure in healthy humans,^61^ highlighting the role of NO signaling in vascular regulation. Nitric oxide also plays an important role in regulating cerebral blood flow and modulating neuronal signaling and neurotransmitter release within the brain, contributing to synaptic plasticity and long-term potentiation, a key cellular mechanism underlying learning and memory.^62^ ^,^ ^63^ During ageing, reductions in NO bioavailability contribute to vascular endothelial dysfunction.^64^ Consistent with this mechanism, we observed enrichment of BH_4_ functional potential in the human microbiota and increased pterin metabolism in the hippocampus of recipient mice. BH_2_ is either reduced to BH₄ through the salvage pathway or recycling pathway.^65^ This upregulation of pterin metabolism aligns with findings from human functional fecal microbiota data, which showed higher BH_4_ functional potential in donors with high SDMT scores. Taken together, this indicates that microbiota-associated metabolic pathways may contribute to the regulation of NO-dependent neurovascular and synaptic processes relevant to cognitive ageing.

BH₄ is a cofactor in tyrosine and tryptophan metabolism, both of which showed differential regulation in recipient mice. Tyrosine is converted to L-DOPA and tryptophan to serotonin.^47^ As found in the microbiota of mice, tyrosine metabolism was also overrepresented, with several metabolites within this cluster enriched in recipient mice receiving FMT from high SDMT donors. Within the hippocampus, the catecholamine biosynthesis pathway appeared to be reduced in recipient mice receiving FMT from high SDMT donors, as indicated by lower levels of 3-Methoxy-4-hydroxy-phenylethyleneglycol. However, the dopamine oxidation pathway appeared to be upregulated in these mice, with enrichment of 2-Carboxy-2,3-dihydro-5,6-dihydroxyindole.

KEGG orthologues associated with *N*-methylhydantoinase were enriched in the microbiota of both humans with high SDMT scores and their recipient mice. This enzyme catalyzes the conversion of *N*-methylhydantoin to *N*-carbamoylsarcosine.^66^ Furthermore, *N*-carbamoylsarcosine was increased in the hippocampus of recipient mice receiving microbiota from donors with higher SDMT scores. There is a need to further explore the role of *N*-carbamoylsarcosine in cognitive function. Sarcosine, a downstream metabolite in this pathway, enhances *N*-methyl-D-aspartate (NMDA) receptor signaling, a pathway involved in synaptic plasticity, learning, and memory.^67^

Altered L-arginine bioavailability and nitric oxide signaling may regulate dopaminergic signaling and influence TH activity, consistent with the altered cortical *Th* expression observed in recipient mice.^68‐71^ Tyrosine metabolism was upregulated, and the *Th* gene was downregulated in the brains of recipient mice receiving FMT from high-SDMT donors. BH_4_ acts as an essential cofactor for TH, the enzyme responsible for catalyzing the rate-limiting step of catecholamine biosynthesis through the conversion of tyrosine to L-DOPA.^47^ ^,^ ^72^ These findings may therefore reflect altered dopaminergic regulation in recipient mice. This is supported by the enrichment of dopamine receptors Drd1, Drd2, and Drd5, which modulate cAMP production and synaptic plasticity.^73^ ^,^ ^74^ The production of cAMP can be stimulated by Drd1 and Drd5 or inhibited by Drd2.^75^ Among these dopaminergic receptors, Drd2 is widely expressed in both neuronal and glial cells and has been implicated in the regulation of neuronal signaling and neuroinflammatory processes.^73^ ^,^ ^76^ ^,^ ^77^ The loss or reduction of Drd2 signaling has been shown to impair hippocampal synaptic plasticity, cognitive function, and hippocampal-dependent associative and spatial learning, increase neuronal vulnerability, and promote neuroinflammatory responses, whereas activation of Drd2 can restore synaptic plasticity and improve cognitive performance in experimental models.^78‐80^ Knockout of Drd5 in mice results in attenuated long-term potentiation in the hippocampus and impaired spatial memory.^81^ Genetic deletion of Drd1 in mice results in altered striatal function, highlighting the role of Drd1 in dopaminergic signaling within basal ganglia circuits.^82^ Drd1 signaling in the mPFC regulates glutamatergic transmission and synaptic plasticity, which in turn influences cognitive function.^83^ Drd5 has a higher affinity for dopamine than Drd1, and as with Drd1, it is highly expressed in the cortex.^84^ ^,^ ^85^ Reduced expression of Drd5 in the prefrontal cortex of rats also induced learning and memory deficits in associative learning and spatial memory, coupled to alterations in neuronal oscillations.^86^ Ppp1r1b is a key intracellular integrator of dopaminergic signaling and is expressed in dopamine receptor–rich neurones, with measurable expression in cortical and hippocampal regions.^87^ The enrichment of Ppp1r1b in high-SDMT recipients therefore provides additional support for altered dopaminergic signaling pathways associated with learning and synaptic plasticity. The dopaminergic system contributes to multiple aspects of brain function, including cognition and executive control.^88^ Together, enrichment of dopamine receptors suggests enhanced dopaminergic responsiveness and balanced modulation of downstream signaling pathways involved in synaptic plasticity and may also contribute to the increased neuronal density detected in recipient mice.

This study has several strengths. Firstly, the discovery analysis was conducted in a large ageing cohort of 867 human participants, providing substantial statistical power to identify associations between the gut microbiome and cognitive performance. Secondly, the microbial taxa and functional pathways identified in the human cohort were consistently observed in recipient mice, and these microbial functions were further reflected in metabolic alterations within the mouse brain. This cross-species consistency strengthens the evidence linking microbiota-associated metabolic pathways to host neurobiological processes. However, several limitations should also be considered. The human cohort was derived from a single geographical population in Spain, which may limit the generalizability of the findings as gut microbiome compositions are known to differ across populations from different geographical regions, with differences in genetics, diet, and lifestyle all playing a role.^89^ Furthermore, microbiota analysis of humans and mice is based on a single timepoint which may not fully capture the temporal dynamics of the gut microbiome.^90^ Future studies across diverse populations and independent cohorts, incorporating longitudinal studies and repeated microbial sampling will be important to validate the microbiome signatures associated with cognitive ageing identified in this study. A further limitation is the inclusion of only male donors and male recipient mice. Therefore, it remains unclear whether the observed microbiome-associated effects would also occur in females, particularly given previously reported sex differences in gut microbiome composition.^91^ ^,^ ^92^ Larger donor cohorts and inclusion of female donors would help to further evaluate the robustness of these microbiome features associated with cognitive ageing. Additionally, although the FMT model provides an experimental framework to investigate potential causal relationships between the microbiota and host physiology, species differences between humans and mice may influence microbiota engraftment and host responses. Mechanistic intervention studies targeting specific microbial taxa or metabolic pathways will be important to further clarify the role of microbiota-derived metabolites in cognitive ageing.

## Conclusion

Both compositional and functional differences in the microbiome were associated with the SDMT score of ageing human donors, and the procedure of FMT has shown that the transfer of microbiota is able to influence the cognitive capabilities of recipient mice via the gut‒brain axis. Microbial upregulation of BH_4_ and L-arginine is suggested to increase L-arginine levels and the production of the vasodilator NO in the hippocampus. Furthermore, recipient mice showed altered dopaminergic signaling, which can be associated with changes in learning and synaptic plasticity. Compared with FMT from low SDMT donors, FMT from high SDMT donors was associated with higher neuronal density in both the cortex and CA1 regions of the brain of recipient mice, along with improved cognitive function. Therapeutic advances for the maintenance of healthy gut microbiota during ageing may therefore promote the production of metabolites involved in arginine, proline, tyrosine and folate pathways. These pathways may help prevent vascular endothelial dysfunction, modulating NO in the brain in addition to regulating the dopaminergic synapses, catecholamine biosynthesis and synaptic plasticity.

## Supplementary Material

Supplementary_figure_4.png Supplementary_figure_4.png

Supplementary_figure_1.png Supplementary_figure_1.png

Supplementary_Figure_2.png Supplementary_Figure_2.png

Supplementary_figure_3.png Supplementary_figure_3.png

Supplemental Material Supplementary_Tables_-_Copy_18_Oct_2026_22_04_AU.xls

Supplementary_data.docx Supplemental Material

## Data Availability

The datasets that support the study's findings are available from the corresponding authors upon reasonable request. The raw metagenomics sequence data derived from human samples in the Aging Imageomics cohorts has been deposited in the European Nucleotide Archive (ENA) under project number PRJEB104496.

## References

[1] World Health Organization . Ageing [Internet]. 2025. Geneva: World Health Organization [cited 2026 May 21]. Available from: https://www.who.int/news-room/fact-sheets/detail/ageing-and-health.

[2] Fjell AM , Walhovd KB . Structural brain changes in aging: courses, causes and cognitive consequences. Rev Neurosci. 2010;21(3):187–221. doi: 10.1515/revneuro.2010.21.3.187. 20879692

[3] Wardlaw JM , Valdés Hernández MC , Muñoz-Maniega S . What are White matter hyperintensities made of? Relevance to vascular cognitive impairment. J Am Heart Assoc. 2015;4(6):e001140. doi: 10.1161/JAHA.114.001140. PMC4599520 26104658

[4] Cox SR , Ritchie SJ , Tucker-Drob EM , Liewald DC , Hagenaars SP , Davies G , Wardlaw JM , Gale CR , Bastin ME , Deary IJ . Ageing and brain White matter structure in 3,513 UK biobank participants. Nat Commun. 2016 Dec 15;7: 13629. doi: 10.1038/ncomms13629. PMC5172385 27976682

[5] Bartsch T , Wulff P . The hippocampus in aging and disease: from plasticity to vulnerability. Neuroscience. 2015;309:1–16. doi: 10.1016/j.neuroscience.2015.07.084. 26241337

[6] Kiely KM , Butterworth P , Watson N , Wooden M . The symbol digit modalities test: normative data from a large nationally representative sample of Australians. Arch Clin Neuropsychol. 2014;29(8):767–775. doi: 10.1093/arclin/acu055. 25352087

[7] Pascoe M , Alamri Y , Dalrymple-Alford J , Anderson T , MacAskill M . The symbol-digit modalities test in mild cognitive impairment: evidence from Parkinson's disease patients. Eur Neurol. 2018;79(3-4):206–210. doi: 10.1159/000485669. 29597229

[8] Smith A . Symbol Digit Modalities Test (SDMT) Manual. 1973. Los Angeles, CA: Western Psychological Services.

[9] Benedict RHB , DeLuca J , Phillips G , LaRocca N , Hudson LD , Rudick R . Validity of the symbol digit modalities test as a cognition performance outcome measure for multiple sclerosis. Mult Scler. 2017;23(5):721–733. doi: 10.1177/1352458517690821. PMC5405816 28206827

[10] Strober L , DeLuca J , Benedict RHB , Jacobs A , Cohen JA , Chiaravalloti N , Hudson LD , Rudick RA , LaRocca NG . Symbol digit modalities test: a valid clinical trial endpoint for measuring cognition in multiple sclerosis. Mult Scler J. 2019;25(13):1781–1790. doi: 10.1177/1352458518808204. PMC6826875 30334474

[11] Grothe M , Domin M , Hoffeld K , Nagels G , Lotze M . Functional representation of the symbol digit modalities test in relapsing-remitting multiple sclerosis. Mult Scler Relat Disord. 2020;43:102159. doi: 10.1016/j.msard.2020.102159. 32473564

[12] Christodoulou C , Krupp LB , Liang Z , Huang W , Melville P , Roque C , Scherl WF , Morgan T , MacAllister WS , Li L , et al. Cognitive performance and MR markers of cerebral injury in cognitively impaired MS patients. Neurology. 2003;60(11):1793–1798. doi: 10.1212/01.WNL.0000072264.75989.B8. 12796533

[13] Geiger AR , Guevara JE , King JB , Hoffman JM , Duff K . Using neuropsychological test scores to predict beta-amyloid deposition in older adults across the late-life cognitive continuum. J Clin Exp Neuropsychol. 2025;47(7):692–700. doi: 10.1080/13803395.2025.2578345. PMC12831542 41137556

[14] Fellows RP , Schmitter-Edgecombe M . Symbol digit modalities test: regression-based normative data and clinical utility. Arch Clin Neuropsychol. 2020;35(1):105–115. doi: 10.1093/arclin/acz020. 31329813

[15] Ghosh TS , Shanahan F , O'Toole PW . The gut microbiome as a modulator of healthy ageing. Nat Rev Gastroenterol Hepatol. 2022;19(9):565–584. doi: 10.1038/s41575-022-00605-x. PMC9035980 35468952

[16] Castells-Nobau A , Fumagalli A , Del Castillo-Izquierdo Á , Rosell-Díaz M , De la Vega-Correa L , Samulėnaitė S , Motger-Albertí A , Arnoriaga-Rodríguez M , Garre-Olmo J , Puig J , et al. Gut microbial modulation of 3-hydroxyanthranilic acid and dopaminergic signalling influences attention in obesity. Gut. 2025;75:705–724. doi: 10.1136/gutjnl-2025-336391. PMC13018844 41015495

[17] Fumagalli A , Castells-Nobau A , Trivedi D , Garre-Olmo J , Puig J , Ramos R , Ramió-Torrentà L , Pérez-Brocal V , Moya A , Swann J , et al. Archaea methanogens are associated with cognitive performance through the shaping of gut microbiota, butyrate and histidine metabolism. Gut Microbes. 2025;17(1):2455506. doi: 10.1080/19490976.2025.2455506. PMC11810085 39910065

[18] Cryan JF , O'Riordan KJ , Sandhu K , Peterson V , Dinan TG . The gut microbiome in neurological disorders. Lancet Neurol. 2020;19(2):179–194. doi: 10.1016/S1474-4422(19)30356-4. 31753762

[19] Procházková N , Laursen MF , La Barbera G , Tsekitsidi E , Jørgensen MS , Rasmussen MA , Raes J , Licht TR , Dragsted LO , Roager HM . Gut physiology and environment explain variations in human gut microbiome composition and metabolism. Nat Microbiol. 2024;9(12):3210–3225. doi: 10.1038/s41564-024-01856-x. PMC11602727 39604623

[20] Claesson MJ , Jeffery IB , Conde S , Power SE , O'Connor EM , Cusack S , O’Connor EM , Harris HMB , Coakley M , Lakshminarayanan B , et al. Gut microbiota composition correlates with diet and health in the elderly. Natur. 2012;488(7410):178–184. doi: 10.1038/nature11319. 22797518

[21] Connell E , Le Gall G , Pontifex MG , Sami S , Cryan JF , Clarke G , Müller M , Vauzour D . Microbial-derived metabolites as a risk factor of age-related cognitive decline and dementia. Mol Neurodegener. 2022;17:43. doi: 10.1186/s13024-022-00548-6. PMC9204954 35715821

[22] Wang HX , Wang YP . Gut microbiota-brain axis. Chin Med J (Engl). 2016;129(19):2373–2380. doi: 10.4103/0366-6999.190667. PMC5040025 27647198

[23] Castells-Nobau A , Puig I , Motger-Albertí A , de la Vega-Correa L , Arnoriaga-Rodríguez M , Mayneris-Perxachs J , Rosell-Díaz M , Escrichs A , Garre-Olmo J , Ramos R , et al. Microviridae bacteriophages influence behavioural hallmarks of food addiction via tryptophan and tyrosine signalling pathways. Nat Metab. 2024;6(11):2157–2186. doi: 10.1038/s42255-024-01157-x. 39587339

[24] Mayneris-Perxachs J , Castells-Nobau A , Arnoriaga-Rodríguez M , Garre-Olmo J , Puig J , Ramos R , Martínez-Hernández F , Burokas A , Coll C , Moreno-Navarrete JM , et al. Caudovirales bacteriophages are associated with improved executive function and memory in flies, mice, and humans. Cell Host Microbe. 2022;30(3):340–356.e8. doi: 10.1016/j.chom.2022.01.013. 35176247

[25] Gheorghe CE , Ritz NL , Martin JA , Wardill HR , Cryan JF , Clarke G . Investigating causality with fecal microbiota transplantation in rodents: applications, recommendations and pitfalls. Gut Microbes. 2021;13(1):1941711. doi: 10.1080/19490976.2021.1941711. PMC8331043 34328058

[26] Puig J , Biarnes C , Pedraza S , Vilanova JC , Pamplona R , Fernández-Real JM , Brugada R , Ramos R , Coll-de-Tuero G , Calvo-Perxas L , et al. The aging imageomics study: rationale, design and baseline characteristics of the study population. Mech Ageing Dev. 2020;189:111257. doi: 10.1016/j.mad.2020.111257. 32437737

[27] Chen S , Zhou Y , Chen Y , Gu J . Fastp: an ultra-fast all-in-one FASTQ preprocessor. Bioinf. 2018;34(17 Suppl 1):i884–i890. doi: 10.1093/bioinformatics/bty560. PMC6129281 30423086

[28] Langmead B , Salzberg SL . Fast gapped-read alignment with bowtie 2. Nat Methods. 2012;9(4):357–359. doi: 10.1038/nmeth.1923. PMC3322381 22388286

[29] Tamames J , Puente-Sánchez F . SqueezeMeta, a highly portable, fully automatic metagenomic analysis pipeline. Front Microbiol. 2019;10:3349. doi: 10.3389/fmicb.2018.03349. PMC6353838 30733714

[30] Liu CM , Luo R , Lam TW . MEGAHIT: an ultra-fast single-node solution for large and complex metagenomics assembly via succinct de bruijn graph. Bioinf. 2015;31(10):1674–1676. doi: 10.1093/bioinformatics/btv033. 25609793

[31] Hyatt D , Chen GL , LoCascio PF , Land ML , Larimer FW , Hauser LJ . Prodigal: prokaryotic gene recognition and translation initiation site identification. BMC Bioinf. 2010;11:119. doi: 10.1186/1471-2105-11-1. PMC2848648 20211023

[32] Buchfink B , Reuter K , Drost HG . Sensitive protein alignments at tree-of-life scale using DIAMOND. Nat Methods. 2021;18(4):366–368. doi: 10.1038/s41592-021-01101-x. PMC8026399 33828273

[33] Kanehisa M . Goto S. Kegg: kyoto encyclopedia of genes and genomes. Nucleic Acids Res. 2000;28(1):27–30. doi: 10.1093/nar/28.1.27. PMC102409 10592173

[34] R Core Team . R: A Language and Environment for Statistical Computing [Internet]. 2025. Vienna, Austria: R Foundation for Statistical Computing [cited 2026 May 29]. Available from: https://www.R-project.org/.

[35] du Sert NP , Hurst V , Ahluwalia A , Alam S , Avey MT , Baker M , Percie du Sert N , Browne WJ , Clark A , Cuthill IC , et al. The ARRIVE guidelines 2.0: updated guidelines for reporting animal research. PLoS Biol. 2020;18(7):e3000410. doi: 10.1371/journal.pbio.3000410. PMC7360023 32663219

[36] Vorhees CV , Williams MT . Morris water maze: procedures for assessing spatial and related forms of learning and memory. Nat Protoc. 2006;1(2):848–858. doi: 10.1038/nprot.2006.116. PMC2895266 17406317

[37] Dobin A , Davis CA , Schlesinger F , Drenkow J , Zaleski C , Jha S , Batut P , Chaisson M , Gingeras TR . STAR: ultrafast universal RNA-seq aligner. Bioinf. 2013;29(1):15–21. doi: 10.1093/bioinformatics/bts635. PMC3530905 23104886

[38] Liao Y , Smyth GK , Shi W . FeatureCounts: an efficient general purpose program for assigning sequence reads to genomic features. Bioinf. 2014;30(7):923–930. doi: 10.1093/bioinformatics/btt656. 24227677

[39] Lin H , Peddada SD . Analysis of compositions of microbiomes with bias correction. Nat Commun. 2020;11(1):3514. doi: 10.1038/s41467-020-17041-7. PMC7360769 32665548

[40] Carvajal-Rodríguez A , de Uña-Alvarez J , Rolán-Alvarez E . A new multitest correction (SGoF) that increases its statistical power when increasing the number of tests. BMC Bioinf. 2009;10:209. doi: 10.1186/1471-2105-10-209. PMC2719628 19586526

[41] Wu T , Hu E , Xu S , Chen M , Guo P , Dai Z , Feng T , Zhou L , Tang W , Zhan L , et al. clusterProfiler 4.0: a universal enrichment tool for interpreting omics data. Innovation (Camb). 2021;2(3):100141. doi: 10.1016/j.xinn.2021.100141. PMC8454663 34557778

[42] Palarea-Albaladejo J , Martín-Fernández JA . ZCompositions - R package for multivariate imputation of left-censored data under a compositional approach. Chemom Intell Lab Syst. 2015;143:85–96. doi: 10.1016/j.chemolab.2015.02.019.

[43] Dieterle F , Ross A , Schlotterbeck G , Senn H . Probabilistic quotient normalization as robust method to account for dilution of complex biological mixtures. Application in 1H NMR metabonomics. Anal Chem. 2006;78(13):4281–4290. doi: 10.1021/ac051632c. 16808434

[44] Ritchie ME , Phipson B , Wu D , Hu Y , Law CW , Shi W , Smyth GK . Limma powers differential expression analyses for RNA-sequencing and microarray studies. Nucleic Acids Res. 2015;43(7):e47–e47. doi: 10.1093/nar/gkv007. PMC4402510 25605792

[45] Robinson MD , McCarthy DJ , Smyth GK . edgeR: a bioconductor package for differential expression analysis of digital gene expression data. Bioinf. 2010;26(1):139–140. doi: 10.1093/bioinformatics/btp616. PMC2796818 19910308

[46] Eichwald T , da Silva LB , Staats Pires AC , Niero L , Schnorrenberger E , Filho CC , Espíndola G , Huang W , Guillemin GJ , Abdenur JE , et al. Tetrahydrobiopterin: beyond its traditional role as a cofactor. Antioxidants (Basel). 2023;12(5):1037. doi: 10.3390/antiox12051037. PMC10215290 37237903

[47] Molinoff PB , Axelrod J . Biochemistry of catecholamines. Annu Rev Biochem. 1971;40:465–500. doi: 10.1146/annurev.bi.40.070171.002341. 4399447

[48] Neves SR , Ram PT , Iyengar R . G protein pathways. Sci. 2002;296(5573):1636–1639. doi: 10.1126/science.1071550. 12040175

[49] Ragonnaud E , Biragyn A . Gut microbiota as the key controllers of “healthy” aging of elderly people. Immun Ageing. 2021;18:2. doi: 10.1186/s12979-020-00213-w. PMC7784378 33397404

[50] Salazar N , González S , Nogacka AM , Rios-Covián D , Arboleya S , Gueimonde M , Reyes-Gavilán CG , et al. Microbiome: effects of ageing and diet. Curr Issues Mol Biol. 2019;36:33–62. doi: 10.21775/cimb.036.033. 31558686

[51] D'Amato A , Di Cesare Mannelli L , Lucarini E , Man AL , Le Gall G , Branca JJV , D’Amato A , Ghelardini C , Amedei A , Bertelli E , et al. Faecal microbiota transplant from aged donor mice affects spatial learning and memory via modulating hippocampal synaptic plasticity and neurotransmission-related proteins in young recipients. Microbiome. 2020;8(1):140. doi: 10.1186/s40168-020-00914-w. PMC7532115 33004079

[52] Li M , Ren J , Bao Y , Wei W , Yu X , He X , gulisima M , Sheng L , Zheng N , Wan J , et al. Aged gut microbiota contributes to cognitive impairment and hippocampal synapse loss in mice. Aging cell. 2025;24(7):e70064. doi: 10.1111/acel.70064. PMC12266779 40219707

[53] Boehme M , Guzzetta KE , Bastiaanssen TFS , van de Wouw M , Moloney GM , Gual-Grau A , Spichak S , Olavarría-Ramírez L , Fitzgerald P , Morillas E , et al. Microbiota from young mice counteracts selective age-associated behavioral deficits. Nat Aging. 2021;1(8):666–676. doi: 10.1038/s43587-021-00093-9. 37117767

[54] Zhu Y , Chen B , Zhang X , Akbar MT , Wu T , Zhang Y , Zhi L , Shen Q . Exploration of the muribaculaceae family in the gut microbiota: diversity, metabolism, and function. Nutrients. 2024;16(16):2660. doi: 10.3390/nu16162660. PMC11356848 39203797

[55] He J , Hou T , Wang Q , Wang Q , Jiang Y , Chen L , Xu J , Qi Y , Jia D , Gu Y , et al. L-arginine metabolism ameliorates age-related cognitive impairment by Amuc_1100-mediated gut homeostasis maintaining. Aging cell. 2024;23(4):e14081. doi: 10.1111/acel.14081. PMC11019123 38236004

[56] Duan D , Wang M , Han J , Li M , Wang Z , Zhou S , Xin W . Advances in multi-omics integrated analysis methods based on the gut microbiome and their applications. Front Microbiol. 2024;15:1509117. doi: 10.3389/fmicb.2024.1509117. PMC11739165 39831120

[57] Visconti A , Le Roy CI , Rosa F , Rossi N , Martin TC , Mohney RP , Li W , de Rinaldis E , Bell JT , Venter JC , et al. Interplay between the human gut microbiome and host metabolism. Nat Commun. 2019;10(1):4505. doi: 10.1038/s41467-019-12476-z. PMC6776654 31582752

[58] Kwon NS , Nathan CF , Stuehr DJ . Reduced biopterin as a cofactor in the generation of nitrogen oxides by murine macrophages. J Biol Chem. 1989;264(34):20496–20501. doi: 10.1016/S0021-9258(19)47089-0. 2584226

[59] Tayeh MA , Marletta MA . Macrophage oxidation of L-arginine to nitric oxide, nitrite, and nitrate: tetrahydrobiopterin is required as a cofactor. J Biol Chem. 1989;264(33):19654–19658. doi: 10.1016/S0021-9258(19)47163-9. 2584186

[60] Mone P , Pansini A , Jankauskas SS , Varzideh F , Kansakar U , Lombardi A , Trimarco V , Frullone S , Santulli G . L-arginine improves cognitive impairment in hypertensive frail older adults. Front Cardiovasc Med. 2022;9:868521. doi: 10.3389/fcvm.2022.868521. PMC9039514 35498050

[61] Bode-Böger SM , Böger RH , Galland A , Tsikas D , Frölich JC . L-arginine-induced vasodilation in healthy humans: pharmacokinetic-pharmacodynamic relationship. Br J Clin Pharmacol. 1998;46(5):489–497. doi: 10.1046/j.1365-2125.1998.00803.x. PMC1873701 9833603

[62] Toda N , Ayajiki K , Okamura T . Cerebral blood flow regulation by nitric oxide: recent advances. Pharmacol Rev. 2009;61(1):62–97. doi: 10.1124/pr.108.000547. 19293146

[63] Prast H , Philippu A . Nitric oxide as modulator of neuronal function. Prog Neurobiol. 2001;64(1):51–68. doi: 10.1016/S0301-0082(00)00044-7. 11250062

[64] Sun HJ , Wu ZY , Nie XW , Bian JS . Role of endothelial dysfunction in cardiovascular diseases: the link between inflammation and hydrogen sulfide. Front Pharmacol. 2020;10:1568. doi: 10.3389/fphar.2019.01568. PMC6985156 32038245

[65] Werner ER , Blau N , Thöny B . Tetrahydrobiopterin: biochemistry and pathophysiology. Biochem J. 2011;438(3):397–414. doi: 10.1042/BJ20110293. 21867484

[66] Kim JM , Shimizu S , Yamada H . Amidohydrolysis of N-methylhydantoin coupled with ATP hydrolysis. 1987 Feb 13;142(3):1006–1012. doi: 10.1016/0006-291X(87)91514-2. 3827889

[67] Martina M , Gorfinkel Y , Halman S , Lowe JA , Periyalwar P , Schmidt CJ , Bergeron R . Glycine transporter type 1 blockade changes NMDA receptor-mediated responses and LTP in hippocampal CA1 pyramidal cells by altering extracellular glycine levels. J Physiol. 2004;557(2):489–500. doi: 10.1113/jphysiol.2004.063321. PMC1665089 15064326

[68] Kiss JP , Hennings EC , Zsilla G , Vizi ES . A possible role of nitric oxide in the regulation of dopamine transporter function in the striatum. Neurochem Int. 1999;34(4):345–350. doi: 10.1016/S0197-0186(99)00019-4. 10372921

[69] West AR , Galloway MP , Grace AA . Regulation of striatal dopamine neurotransmission by nitric oxide: effector pathways and signaling mechanisms. Synapse. 2002;44(4):227–245. doi: 10.1002/syn.10076. 11984858

[70] Wang Y , Sung CC , Chung KKK . Novel enhancement mechanism of tyrosine hydroxylase enzymatic activity by nitric oxide through S-nitrosylation. Sci Rep. 2017 Mar 13;7: 44154. doi: 10.1038/srep44154. PMC5347018 28287127

[71] Zhu XZ , Luo LG . Effect of nitroprusside (nitric oxide) on endogenous dopamine release from rat striatal slices. J Neurochem. 1992;59(3):932–935. doi: 10.1111/j.1471-4159.1992.tb08332.x. 1494918

[72] Daubner SC , Le T , Wang S . Tyrosine hydroxylase and regulation of dopamine synthesis. Arch Biochem Biophys. 2011;508(1):1–12. doi: 10.1016/j.abb.2010.12.017. PMC3065393 21176768

[73] Beaulieu JM , Gainetdinov RR . The physiology, signaling, and pharmacology of dopamine receptors. Pharmacol Rev. 2011;63(1):182–217. doi: 10.1124/pr.110.002642. 21303898

[74] Girault JA , Greengard P . The neurobiology of dopamine signaling. Arch Neurol. 2004;61(5):614–644. doi: 10.1001/archneur.61.5.641. 15148138

[75] Missale C , Nash SR , Robinson SW , Jaber M , Caron MG . Dopamine receptors: from structure to function. Physiol Rev. 1998;78(1):189–225. doi: 10.1152/physrev.1998.78.1.189. 9457173

[76] Sun H , Xie X , Li Y , Zhu W , Hou Y , Xie H , Zhou B , Zhang J , Wang Q , Liu J , et al. Function and mechanism of dopamine receptor D2 (DRD2) in neuroinflammation and behavioral disorders in C57BL/6J mice infected with *toxoplasma gondii* TgCtWh6 strain. Brain Behav Immun. 2026;131:106136. doi: 10.1016/j.bbi.2025.106136. 41106615

[77] Rangel-Barajas C , Silva I , López-Santiago LM , Aceves J , Erlij D , Florán B . L-DOPA-induced dyskinesia in hemiparkinsonian rats is associated with up-regulation of adenylyl cyclase type V/VI and increased GABA release in the substantia nigra reticulata. Neurobiol Dis. 2011;41(1):51–61. doi: 10.1016/j.nbd.2010.08.018. 20736067

[78] Shao W , Zhang SZ , Tang M , Zhang XH , Zhou Z , Yin YQ , Huang Y , Liu Y , Wawrousek E , Chen T , et al. Suppression of neuroinflammation by astrocytic dopamine D2 receptors via αB-crystallin. Natur. 2013;494(7435):90–94. doi: 10.1038/nature11748. 23242137

[79] Espadas I , Ortiz O , Garcia-Sanz P , Sanz-Magro A , Alberquilla S , Solis O , García-Sanz P , Delgado-García JM , Gruart A , Moratalla R . Dopamine D2R is required for hippocampal-dependent memory and plasticity at the CA3-CA1 synapse. Cereb Cortex. 2021;31(4):2187–2204. doi: 10.1093/cercor/bhaa354. PMC7945019 33264389

[80] Gao H , Zhang Y , Luo D , Xu J , Tan S , Li Y , Qi W , Zhai Q , Wang Q . Activation of the hippocampal DRD2 alleviates neuroinflammation, synaptic plasticity damage and cognitive impairment after sleep deprivation. Mol Neurobiol. 2023;60(12):7208–7221. doi: 10.1007/s12035-023-03514-5. 37543530

[81] Moraga-Amaro R , González H , Ugalde V , Donoso-Ramos JP , Quintana-Donoso D , Lara M , Morales B , Rojas P , Pacheco R , Stehberg J . Dopamine receptor D5 deficiency results in a selective reduction of hippocampal NMDA receptor subunit NR2B expression and impaired memory. Neuropharmacology. 2016;103:222–235. doi: 10.1016/j.neuropharm.2015.12.018. 26714288

[82] Drago J , Gerfen CR , Lachowicz JE , Steiner H , Hollon TR , Love PE , Ooi GT , Grinberg A , Lee EJ , Huang SP . Altered striatal function in a mutant mouse lacking D1A dopamine receptors. Proc Natl Acad Sci U S A. 1994;91(26):12564–12568. doi: 10.1073/pnas.91.26.12564. PMC45479 7809078

[83] Yin Y , Hu J , Wu H , Yang X , Qi J , Huang L , Luo Z , Jin S , Chen H , Li X , et al. Astrocytic dopamine D1 receptor modulates glutamatergic transmission and synaptic plasticity in the prefrontal cortex through D-serine. Acta Pharm Sin B. 2025;15(9):4692–4710. doi: 10.1016/j.apsb.2025.07.034. PMC12491719 41049738

[84] Sunahara RK , Guan HC , O'Dowd BF , Seeman P , Laurier LG , Ng G , George SR , Torchia J , Van Tol HHM , Niznik HB . Cloning of the gene for a human dopamine D5 receptor with higher affinity for dopamine than D1. Natur. 1991;350(6319):614–619. doi: 10.1038/350614a0. 1826762

[85] Bordelon-Glausier JR , Khan ZU , Muly EC . Quantification of D1 and D5 dopamine receptor localization in layers I, III, and V of macaca mulatta prefrontal cortical area 9: coexpression in dendritic spines and axon terminals. J Comp Neurol. 2008 Jun 20;508(6):893–905. doi: 10.1002/cne.21710. PMC2586172 18399540

[86] Albeely AM , Nolan CJ , Rasmussen DJ , Bailey CDC , Perreault ML . Cortical dopamine D5 receptors regulate neuronal circuit oscillatory activity and memory in rats. CNS Neurosci Ther. 2023;29(9):2469–2480. doi: 10.1111/cns.14210. PMC10401150 37076975

[87] Ouimet CC , LaMantia AS , Goldman-Rakic P , Rakic P , Greengard P . Immunocytochemical localization of DARPP-32, a dopamine- and cyclic AMP-regulated phosphoprotein, in the primate brain. J Comp Neurol. 1992;323(2):209–218. doi: 10.1002/cne.903230206. 1328330

[88] Ott T , Nieder A . Dopamine and cognitive control in prefrontal cortex. Trends Cogn Sci. 2019;23(3):213–234. doi: 10.1016/j.tics.2018.12.006. 30711326

[89] Parizadeh M , Arrieta MC . The global human gut microbiome: genes, lifestyles, and diet. Trends Mol Med. 2023;29:789–801. doi: 10.1016/j.molmed.2023.07.002. 37516570

[90] Vandeputte D , De Commer L , Tito RY , Kathagen G , Sabino J , Vermeire S , Faust K , Raes J . Temporal variability in quantitative human gut microbiome profiles and implications for clinical research. Nat Commun. 2021;12(1):6740. doi: 10.1038/s41467-021-27098-7. PMC8602282 34795283

[91] Luan Z , Fu S , Qi S , Li C , Chen J , Zhao Y , Zhang H , Wu J , Jing Y , Wang S , et al. A metagenomics study reveals the gut microbiome as a sex-specific modulator of healthy aging in hainan centenarians. Exp Gerontol. 2024;186:112356. doi: 10.1016/j.exger.2023.112356. 38185288

[92] Zhang S , Cai H , Wang C , Zhu J , Yu Y . Sex-dependent gut microbiota-brain-cognition associations: a multimodal MRI study. BMC Neurol. 2023;23(1):169. doi: 10.1186/s12883-023-03217-3. PMC10134644 37106317

