# Supplementary material for: Human cognitive performance-associated gut microbiota influences learning and memory in mice through arginine and pterin metabolism
Source: Gut Microbes. 2026 Nov 10;18(1):2750588. doi: 10.1080/19490976.2026.2750588 (PMC13757691; doi:10.1080/19490976.2026.2750588)
Supplement: Supplementary file 1 [file KGMI_A_2750588_SM9373.docx]

**Supplementary data**

**Supplementary Figure 1.** Engraftment analysis from human donors into recipient mice. (a) PCA of CLR-transformed metagenomic species abundance data comparing FMT recipient mice and control mice. (b) Venn diagram showing the shared detected microbial species among human donors, post-FMT recipient mice, and control mice. (c) Donor-specific species engraftment in recipient mice. For each human donor, the percentage of donor species detected in the corresponding post-FMT recipient mice is shown. Each points represents individual recipient mice and boxplots summarise the distribution for each donor.

**Supplementary Figure 2.** Morris water maze (MWM) tests showing: Time to platform; Path efficiency; Corrected Integrated Path Length (CIPL). Data analysed by including donor SDMT as a continuous factor using a mixed linear model with the interaction between test day and donor SDMT scores. Lines show model-estimated means (shaded 95% confidence intervals).

**Supplementary Figure 3.** Metagenomics analysis of the microbiota and metabolomics of the hippocampus showing data associated with SDMT in the mouse recipients receiving FMT from the selected 13 human donors, controlled for age, BMI, and years of education. (a) Volcano plots showing faecal microbial features associated with donor SDMT following ANCOM-BC analysis of shotgun metagenomics data and correction for multiple comparisons, with the most significant KEGG orthologues labelled. (b) C-net plot of differential microbial genes of the tyrosine metabolic pathway following over-representation KEGG pathway analyses (q value < 0.1) mapping the KEGG orthologues to determine overexpressed metabolic pathways.

**Supplementary Figure 4.** RNA-seq of the cortex in recipient mice controlled for age, BMI, and years of education. Corresponding C-net plot of overexpressed pathways and genes corresponding to the Neuroactive ligand-receptor interaction.
